# Hepatic and pancreatic extracellular volume fraction analysis using contrast-enhanced CT in patients with diabetes mellitus and pre-diabetes

**DOI:** 10.1007/s11604-024-01531-5

**Published:** 2024-02-14

**Authors:** Hideyuki Fukui, Hiromitsu Onishi, Atsushi Nakamoto, Takahiro Tsuboyama, Takashi Ota, Toru Honda, Kengo Kiso, Eriko Yoshidome, Yukihiro Enchi, Mitsuaki Tatsumi, Noriyuki Tomiyama

**Affiliations:** 1 Department of Diagnostic and Interventional Radiology, Osaka University Graduate School of Medicine, D1, 2-2, Yamadaoka, Suita, Osaka 565-0871 Japan; 2 Division of Radiology, Department of Medical Technology, Osaka University Hospital, Suita, Japan; 3 Department of Medical Physics and Engineering, Osaka University Graduate School of Medicine, Suita, Japan

**Keywords:** Diagnostic imaging, Multidetector computed tomography, Contrast media, Fibrosis, Diabetes mellitus

## Abstract

**Purpose:**

Liver and pancreatic fibrosis is associated with diabetes mellitus (DM), and liver fibrosis is associated with pancreatic fibrosis. This study aimed to investigate the relationship between the hepatic and pancreatic extracellular volume fractions (fECVs), which correlate with tissue fibrosis, and their relationships with DM and pre-DM (pDM).

**Material and methods:**

We included 100 consecutive patients with known or suspected liver and/or pancreatic diseases who underwent contrast-enhanced CT. Patients were classified as nondiabetes, pDM, and DM with hemoglobin A1c (HbA1c) levels of < 5.7%, 5.7%–6.5%, and ≥ 6.5% or fasting plasma glucose (FPG) levels of < 100, 100–125 mg/dL, and ≥ 126 mg/dL, respectively. Subtraction images between unenhanced and equilibrium-phase images were prepared. The liver and the pancreas were automatically extracted using a high-speed, three-dimensional image analysis system, and their respective mean CT values were calculated. The enhancement degree of the aorta (Δaorta) was measured. fECV was calculated using the following equation: fECV = (100 − hematocrit) * Δliver or pancreas/Δaorta. Differences were investigated in hepatic and pancreatic fECVs among the three groups, and the correlation between each two in hepatic fECV, pancreatic fECV, and HbA1c was determined.

**Results:**

The pancreatic fECV, which was positively correlated with the hepatic fECV and HbA1c (r = 0.51, *P* < 0.001, and r = 0.51, *P* < 0.001, respectively), significantly differed among the three groups (*P* < 0.001) and was significantly greater in DM than in pDM or nondiabetes and in pDM with nondiabetes (*P* < 0.001). Hepatic fECV was significantly greater in DM than in nondiabetes (*P* < 0.05).

**Conclusion:**

The pancreatic fECV and pDM/DM are closely related.

## Introduction

Diabetes mellitus (DM) is one of the most critical public health challenges of recent years. The International Diabetes Federation data reported that 537 million people (approximately 10% of the world population aged 20–79 years) had DM and 6.7 million died from DM in 2021, and the number of people with DM is expected to increase to 634 million by 2030 and 783 million by 2045 [1].

DM is a risk factor for liver fibrosis in metabolic dysfunction-associated steatotic liver disease (MASLD) [2]. The underlying mechanism of hepatic fibrosis is insulin resistance in adipose tissue that enhances inflammatory cytokine (*e.g.*, tumor necrosis factor-α, monocyte chemotactic protein 1, interleukin-6) production that acts directly on the liver via circulating plasma [3]. Conversely, hepatokine (*e.g.*, Fetuin-A, selenoprotein P) production associated with fibrosis in severe MASLD is mainly involved in the pathogenesis and progression of DM [4]. The American Diabetes Association guidelines recommend that patients with DM and pre-DM (pDM) who have fatty liver or elevated alanine aminotransferase levels should be evaluated for the presence of liver fibrosis [5]. Additionally, pancreatic fibrosis is thought to cause DM because it destroys pancreatic tissue, causing insulin deficiency [6]. Moreover, DM stimulates the expression of α-smooth muscle actin by activating pancreatic stellate cells, causing pancreatic fibrosis [7, 8]. This suggests the existence of a close link between DM and pancreatic fibrosis.

Considering these factors together, pancreatic fibrosis is predicted to progress alongside liver fibrosis. Clarifying the correlation between the progression of pancreatic fibrosis and hepatic fibrosis is vital to elucidate the pathogenesis of organ fibrosis and diabetes. In particular, it will help elucidate the pathogenesis of DM and establish its optimal management in patients with chronic liver diseases.

Hepatic fibrosis is noninvasively evaluated by blood markers, US elastography, and MR elastography [9, 10]. There are many recent studies on liver fibrosis evaluation using extracellular volume fraction (fECV) analysis with contrast-enhanced CT [11–13]. Additionally, the fECV calculated using contrast-enhanced CT is used to noninvasively evaluate pancreatic fibrosis [14, 15]. Fibrosis, which is commonly defined as extracellular matrix protein excess and improper deposition, is often found in diabetic tissues [16, 17]. The fECV, which represents the volume ratio of the extracellular fluid space in tissue, is the difference in tissue and aorta concentrations between the equilibrium and unenhanced phases of contrast-enhanced CT images corrected by the hematocrit value, assuming an even distribution of the extracellular fluid iodine contrast medium in the extracellular fluid cavity in the equilibrium phase.

Changes in the glucose availability, glycation rate, and erythrocyte lifespan alter HbA1c levels, although HbA1c is the gold standard for measuring long-term glycemic control in patients with DM. One or more of these factors are affected and contribute to the reduced HbA1c levels that are inconsistent with the actual severity of DM in patients with cirrhosis or moderate-to-severe anemia [18, 19]. Therefore, in these patients, fECV may be a valuable adjunct to diagnose DM.

Herein, we investigated the relationship between hepatic and pancreatic fECVs and their relationships with DM and pDM. We hypothesized that a significant relationship exists between pancreatic fibrosis progression and hepatic fibrosis progression, and fECV analysis with contrast-enhanced CT can be a valuable tool to evaluate fibrosis in the tissues of patients with diabetes. This study aimed to validate this hypothesis and explore the potential use of fECV analysis with contrast-enhanced CT for evaluating fibrosis in patients with diabetes. By establishing this relationship, this study contributes to the understanding of the pathogenesis of organ fibrosis and diabetes and provides insights for optimal management in patients with chronic liver diseases and DM or pDM.

## Materials and methods

This study was approved by our institutional review board, and informed consent was waived for this retrospective study.

### Patients

**Fig. 1:**
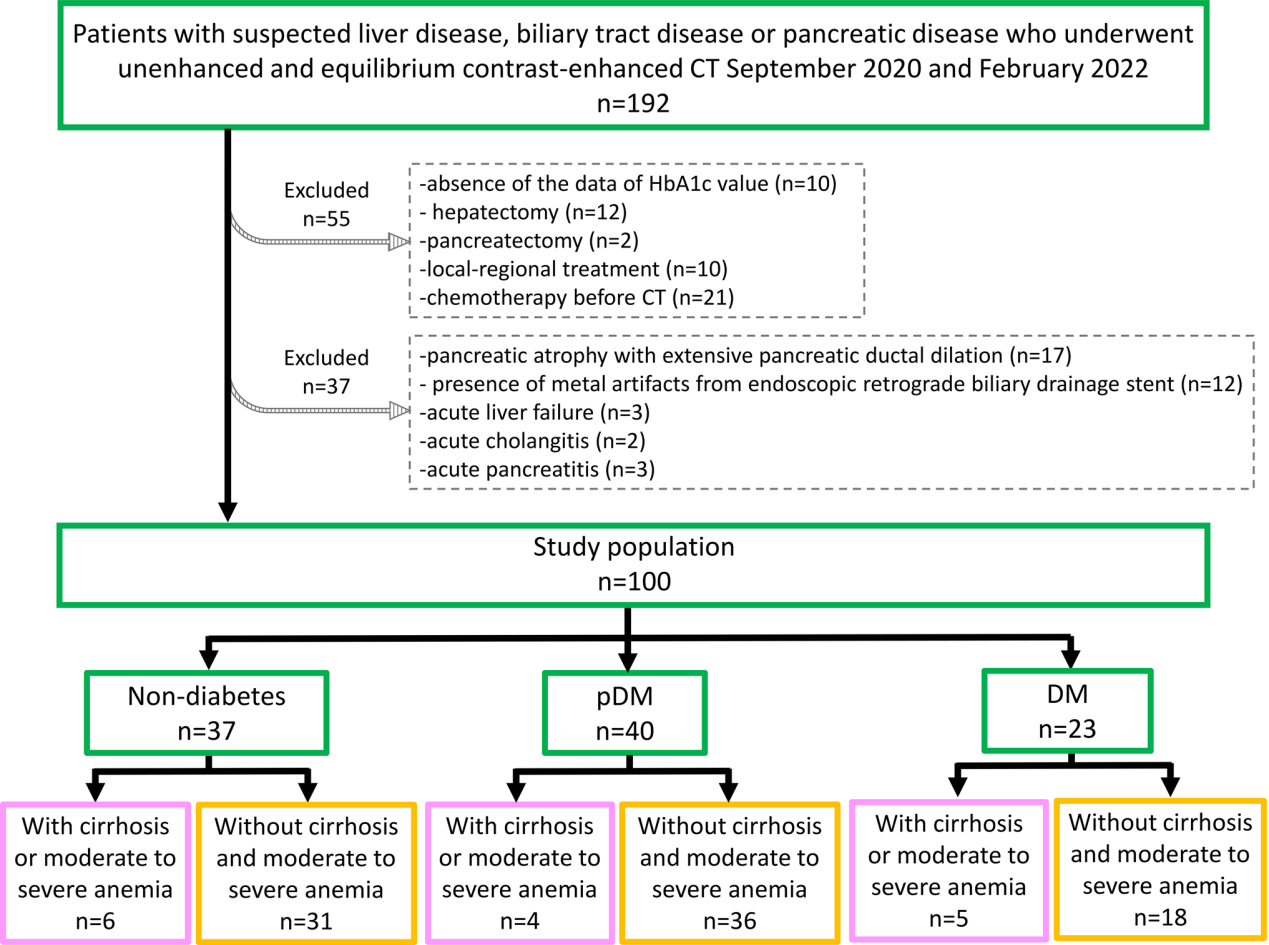
Flowchart of the patient enrollment process

A total of 192 consecutive patients with suspected liver disease, biliary tract disease, or pancreatic disease who underwent unenhanced and equilibrium contrast-enhanced CT from September 2020 to February 2022 at our institution were evaluated. Ten patients who lacked HbA1c data, 12 who underwent hepatectomy, two who underwent pancreatectomy, 10 who underwent local–regional treatment (7 transcatheter arterial chemoembolization and three radiofrequency ablations), and 21 who underwent chemotherapy before CT were excluded. Of the remaining 137 patients, 17 were excluded due to pancreatic atrophy with extensive pancreatic ductal dilation, which made it extremely difficult to automatically extract the pancreas. In addition, 12 patients were excluded owing to the presence of metal artifacts from the endoscopic retrograde biliary drainage stent, and the findings were evaluated by two board-certified radiologists (11 and 26 years of experience, respectively), who assessed the images by consensus. Moreover, three patients with evidence of acute liver failure, two with acute cholangitis, and three with acute pancreatitis, as determined in accordance with established diagnostic imaging or laboratory test guidelines [20–22], were excluded. To ensure the proper acquisition of the equilibrium phase, we compared ΔAorta and ΔPortal in each patient. If substantial differences (over ± 10 HU) were observed between ΔAorta and ΔPortal, these patients were considered outliers and were subsequently excluded from this study. The main intention was to exclude patients meeting the criteria of ΔAorta and ΔPortal (over ± 10 HU). However, no individuals fulfilled these criteria in our sample [13]. Finally, 100 consecutive patients (62 males, 38 females; median age: 62 years; interquartile range: 49.25–73 years) were evaluated. Eighty-four of the 100 study patients were diagnosed either pathologically (62 patients) or through CT scans (22 patients), and their specific diagnoses are detailed in Table 1, which also presents the characteristics of all patients. CT scans for screening or suspected liver or pancreatic tumors were performed in 16 patients; however, they revealed no lesions and made no diagnosis. The patients were divided into three groups per the American Diabetes Association criteria [23] and the average of up to three measurements of HbA1c and fasting blood glucose (FPG) taken before and after the CT scan was used: nondiabetes with HbA1c of < 5.7% or FPG of < 100 mg/dL, pDM with HbA1c of 5.7%–6.4% or FPG of 100–125 mg/dL, and DM with HbA1c of ≥ 6.5% or FPG of ≥ 126 mg/dL. The interval between the nearest measurement of these values and CT ranged from 0 to 83 days (mean: 13.9 ± 16.8 days). Each group was divided into two groups: those with cirrhosis or moderate-to-severe anemia (total, n = 15) and those without cirrhosis and moderate-to-severe anemia (total, n = 85) (Fig. 1). The diagnosis of cirrhosis was based on the clinical spectrum (irregular and nodular liver; other findings included small and shrunken liver, splenomegaly, and complications of cirrhosis) (five patients) or pathological findings (five patients) [24]. The diagnosis of moderate-to-severe anemia was made on the basis of hemoglobin levels and followed the World Health Organization’s criteria [25], with the interval between the nearest measurement of the values and CT ranging from 0 to 45 days (mean: 8.3 ± 10.5 days). Additional patient data were collected from electronic health records and are summarized in Table 2.

**Table 1:** Patients’ characteristics

	Value
Characteristics	
No. of patients	100
Age (years)*	62 (49.25–73)
Female	38 (38%)
Male	62 (62%)
Purpose of undergoing contrast-enhanced CT	
Malignant liver lesion	
Hepatocellular carcinoma	11 (11%)
Liver metastasis	11 (11%)
Colorectal cancer	7 (7%)
Esophageal cancer	3 (3%)
Lung cancer	1 (1%)
Intrahepatic cholangiocarcinoma	7 (7%)
Combined hepatocellular-cholangiocellular carcinoma	1 (1%)
Benign liver lesion	
Liver cyst	3 (3%)
Hepatic hemangioma	17 (17%)
Epithelioid hemangioendothelioma	1 (1%)
Intraductal papillary neoplasm of the bile duct	1 (1%)
Diffuse liver lesion	
Primary biliary cholangitis	2 (2%)
Hepatic sarcoidosis	1 (1%)
Malignant pancreatic lesion	
Pancreatic ductal adenocarcinoma	9 (9%)
Intraductal papillary mucinous carcinoma	2 (2%)
Benign pancreatic lesion	
Intraductal papillary mucinous neoplasm	3 (3%)
Mucinous cystadenoma	3 (3%)
Solid pseudopapillary neoplasm	1 (1%)
Pancreatic neuroendocrine tumor	3 (3%)
Other Conditions	
Duodenal neuroendocrine tumor	2 (2%)
Duodenal adenoma	1 (1%)
Distal cholangiocarcinoma	3 (3%)
Congenital biliary dilatation	1 (1%)
Gallbladder cancer	1 (1%)
For screening for suspected liver or pancreatic tumors	16 (16%)

Unless otherwise indicated, data show the number of patients (n = 100), with percentages in parentheses

*The table displays a median with its interquartile range in parentheses

**Table 2:** Comparison of computed tomography imaging measurements and the clinical characteristics among the three groups

	Nondiabetes	pDM	DM	*P*
No. of patients	37	40	23	
Age (years)	52.0 (45.0–70.0)	63.5 (52.8–74.0)	70.0 (62.0–75.0)	< 0.01
No. of female patients	15	16	7	0.70
No. of male patients	22	24	16	
Hepatic fECV	29.3 (28.1–30.5)	30.1 (28.7–32.3)	33.5 (29.6–35.6)	< 0.05
Pancreatic fECV	29.8 (28.1–31.9)	32.7 (31.5–34.2)	36.6 (35.1–38.9)	< 0.001
Volume of the liver (ml)	1123.7 (1010.5–1305.1)	1168.6 (983.3–1454.8)	1129.6 (882.5–1406.3)	0.68
Volume of the pancreas (ml)	69.1 (56.8–87.1)	65.0 (57.6–88.2)	64.4 (47.3–82.5)	0.58
HbA1c (%)	5.45 (5.30–5.60)	5.82 (5.73–6.12)	6.83 (6.45–7.32)	< 0.001
FPG (mg/dL)	93.0 (89.0–95.0)	106.0 (98.5–111.0)	141.0 (120.0–185.0)	< 0.001
Body mass index	21.6 (19.7–26.5)	21.5 (19.5–23.8)	22.8 (19.3–25.8)	0.55
Hemoglobin (g/dL)	13.1 (12.5–14.7)	13.3 (12.4–14.5)	13.8 (12.6–14.5)	0.96
Hematocrit (%)	40.6 (37.7–43.2)	40.2 (37.3–43.6)	41.8 (37.8–43.7)	0.81
Creatinine (mg/dL)	0.81 (0.68–0.94)	0.7 (0.62–0.76)	0.73 (0.67–0.90)	< 0.05
Cholesterol (mg/dL)	200.0 (178.0–222.3)	183.0 (161.0–217.0)	191.0 (175.0–224.0)	0.29
AST (IU/l)	22.0 (17.0–28.0)	21.0 (16.8–27.3)	27.0 (21.0–56.5)	< 0.05
ALT (IU/l)	19.0 (15.0–35.0)	18.0 (13.0–26.5)	22.0 (21.0–49.0)	0.10
ALP (IU/l)	72.0 (59.0–97.0)	82.0 (64.0–106.0)	98.0 (76.5–132.0)	0.08
γ-GT (IU/l)	35.0 (17.0–123.0)	38.0 (20.0–62.0)	44.0 (26.0–111.5)	0.54
Total bilirubin (mg/dl)	0.70 (0.50–1.10)	0.60 (0.50–0.825)	0.70 (0.60–1.15)	0.34
Albumin (g/dl)	4.30 (4.0–4.50)	4.20 (3.90–4.40)	4.10 (3.80–4.40)	0.49
Total protein (g/dl)	7.20 (6.90–7.40)	7.35 (6.70–7.60)	7.10 (6.93–7.58)	0.79
Triglyceride (mg/dl)	80.0 (63.5–105.0)	96.0 (79.0–127.5)	99.5 (62.5–163.3)	0.17
Prothrombin time (%)	90.0 (84.0–102.0)	92.0 (83.5–101.5)	92.0 (85.5–97.8)	0.98
Chronic liver disease				
Hepatitis B virus	4	2	2	0.13
Hepatitis C virus	0	4	1	
Alcohol	1	0	1	
MASH	3	0	2	

Data are medians with interquartile range in parentheses. *fECV* extracellular volume fraction, *FPG* fasting plasma glucose, *AST* aspartate aminotransferase, *ALT* alanine aminotransferase, *ALP* alkaline phosphatase, *γ-GT* gamma-glutamyl transpeptidase, *pDM* prediabetes, *DM* diabetes mellitus, *MASH* metabolic dysfunction-associated steatohepatitis

### CT imaging

Images were obtained in the early arterial, late arterial, portal, and equilibrium phases in 59 patients, whereas images were obtained in the late arterial, portal, and equilibrium phases in 41 patients. CT imaging using 320-channel (Aquilion One Genesis Edition; Canon Medical Systems, Otawara, Japan), 160-channel (Aquilion Precision; Canon Medical Systems), or 64-channel (Discovery CT 750 HD or Discovery CT 750 HD Freedom Edition; GE Healthcare) devices was performed. The scanning parameters were as follows: collimation: 0.625 × 64 or 0.5 × 80 mm; pitch: 1.375 or 0.813 mm/rotation; rotation time: 0.4 or 0.5 s/rotation; exposure parameters:120 kV and auto-exposure control with a noise index standard deviation of 13.8 or 15; and field of view: 345 mm. Image reconstruction was conducted with a slice thickness of 5 mm, using either an FC03 or GE standard kernel. We assessed patient radiation exposure using the CTDIvol indicator, and the median value was 8.66 mGy. Early arterial phases were obtained 8 s after attaining 100-HU attenuation of the descending aorta (using bolus-tracking technique) after the precontrast scan. Late arterial phases were obtained 7 s after the early arterial phases or 20 s after attaining 100-HU attenuation of the descending aorta (using the bolus-tracking technique). The portal and equilibrium phases were acquired 30 s after the late arterial phase and 120 s after the portal phase. The average time from the start of contrast medium injection to scanning of the equilibrium phase was 204.1 s; 95% confidence interval 188.4–219.8 s; and range 191–225 s. Contrast medium was intravenously administered with an iodine concentration of 600 mg I/kg body weight at a fixed duration of 30 s. Unenhanced and dynamic contrast-enhanced images were obtained; however, only unenhanced and equilibrium-phase images were used. A nonlinear nonrigid registration process (SURE SUBTRACTION Iodine Mapping, Canon Medical Systems, Tokyo, Japan: SSIM) was used to generate subtraction images between unenhanced and equilibrium-phase images were generated [26].

### Quantitative assessment of images

**Fig. 2:**
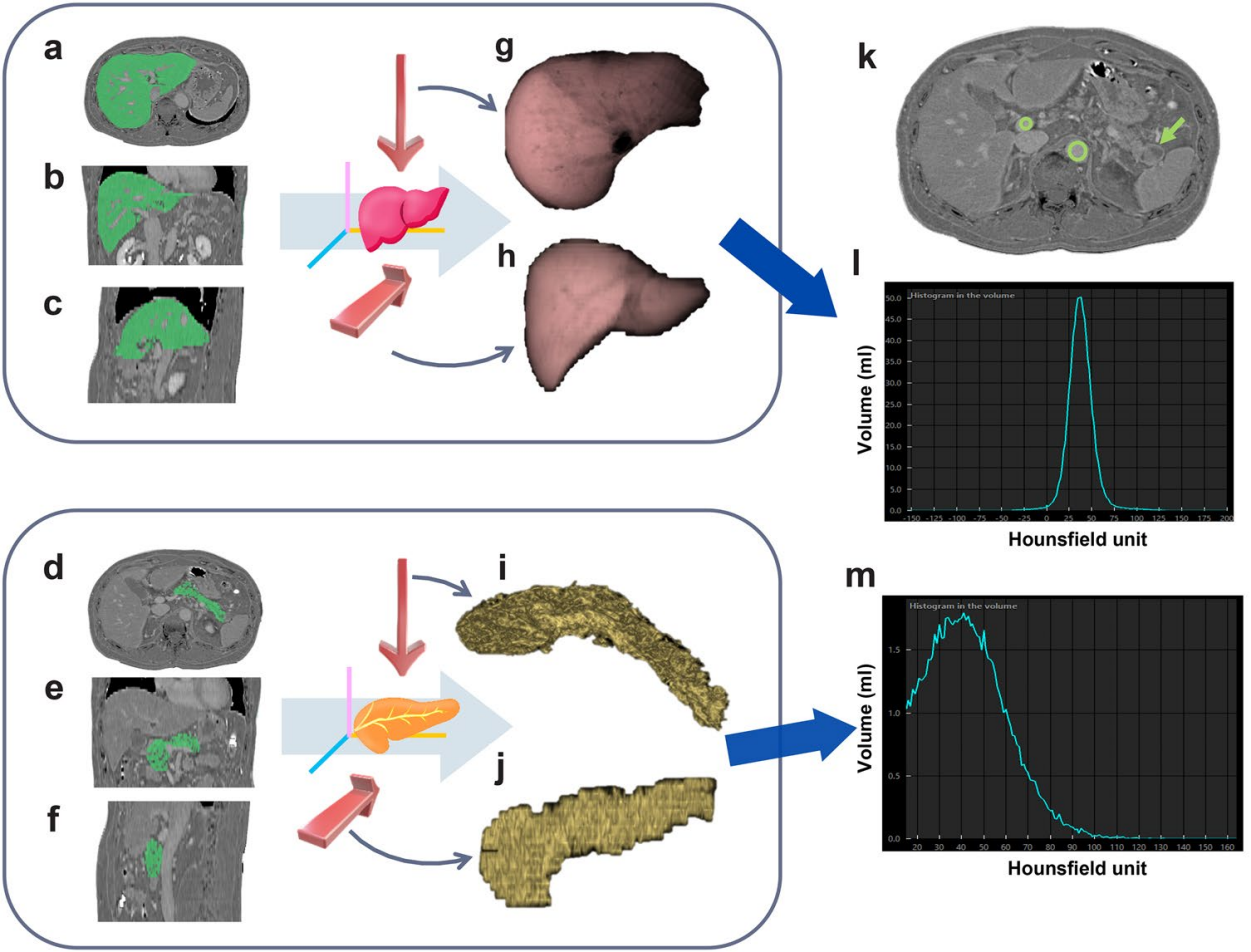
Images of a 63-year-old male with intraductal papillary mucinous adenoma (IPMA) in the prediabetes group (HbA1c: 6.2%, FPG = 107 mg/dL). **a**–**f** The green areas indicate hepatic and pancreatic segmentation on the subtraction images between unenhanced and equilibrium-phase images. **g**–**j** Three-dimensional (3D) volumetry excluded focal lesions, major vascular branches, and ducts. **k** Region of interest locations for the portal vein and aorta on the subtraction image. IPMA in the tail of the pancreas (arrow). **l**, **m** A CT histogram showing the CT value of the green area and its distribution percentage. The mean CT value of the liver and pancreas was 37.7 HU (range: − 155–200 HU) and 43.4 HU (range: 15–164 HU). Hepatic fECV: 29.4%; pancreatic fECV: 33.8% (in this patient)

The fECV measurements were performed on an image workstation (SYNAPSE VINCENT; FUJIFILM Medical Co., Ltd., Tokyo, Japan). The liver and pancreas were automatically extracted, excluding focal lesions, major vascular branches, and ducts, using an application of the workstation on the subtraction images between unenhanced and equilibrium-phase images. Pancreatic fatty infiltration could affect the fECV; therefore, a thresholding process was used by setting a low threshold limit to remove pancreatic fatty infiltration as much as possible while preserving the pancreatic parenchymal tissue to reduce the potential impact of fatty infiltration on fECV measurements. The mean enhancement degree (in Hounsfield units) of the whole liver and whole pancreas parenchyma (ΔLiver and ΔPancreas) were measured. Regions of interest were drawn as large as possible in the lumen of the aorta and portal vein, not including the vessel wall, to measure the enhancement degree of the aorta (ΔAorta) and portal vein (ΔPortal). The hepatic and pancreatic fECV were calculated using the following equation: fECV [%] = (100 − hematocrit [%]) * ΔLiver (or ΔPancreas) / ΔAorta [11, 26]. The hematocrit levels closest to the date of the CT examination were selected for the analysis (median, 4.5 ± 10.4 days; range, 0–45 days). Additionally, we measured the volumes of the liver and pancreas (Fig. 2).

### Statistical analysis

The Shapiro–Wilk test was performed to determine the normality of distribution of the blood test results and the hepatic and pancreatic fECV. Spearman’s rho coefficient (r) was used to assess the degree of association between each two of the three parameters (hepatic fECV, pancreatic fECV, and HbA1c). Fisher’s r-to-z transformation was used to calculate the Z value to evaluate the significance of the difference between the two correlation coefficients. The Kruskal–Wallis test or Fisher’s exact test followed by the Steel–Dwass post hoc test were used to compare the differences in blood test results, hepatic fECV, pancreatic fECV, and HbA1c among the three patient groups. We used the Mann–Whitney *U* test to compare hepatic fECV, pancreatic fECV, and HbA1c between patients with or without cirrhosis and moderate-to-severe anemia. Receiver operating characteristic (ROC) analyses were performed to define the Youden index-based optimal cutoff value for hepatic fECV and pancreatic fECV [27]. The pairwise McNemar test was used to compare the sensitivity and specificity, and DeLong’s test was used to determine the difference between two areas under the curve (AUCs). *P*-values of < 0.05 were considered significant for all tests. The following ranges were used to assess the strength of correlations: r < 0.19, very weak correlation; 0.20–0.39, weak correlation; 0.40–0.59, moderate correlation; between 0.60 and 0.79, a strong correlation; and between 0.80 and 1.00, a very strong correlation. All statistical analyses were performed using EZR version 1.50 (Saitama Medical Center, Jichi Medical University).

## Results

Patient characteristics are shown in Table 1. Of the 100 patients, 37, 40, and 23 were in the nondiabetes, pDM, and DM groups, respectively. HbA1c and FPG differed significantly in each group (*P* < 0.001, respectively). The patients’ CT imaging measurements and clinical conditions for each group are shown in Table 2.

**Fig. 3:**
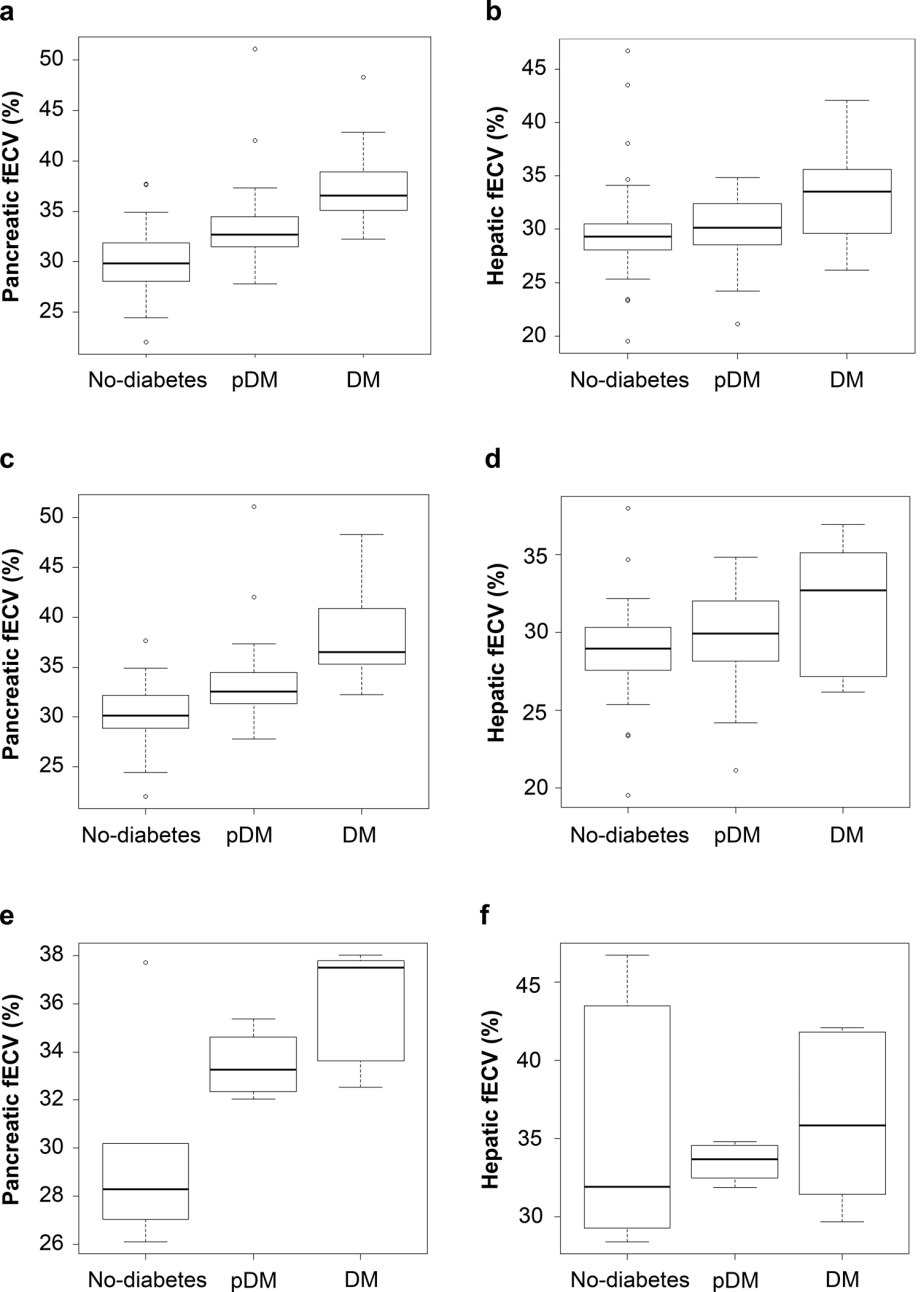
Box plot showing pancreatic and hepatic fECV in the three groups. **a** Pancreatic fECV is significantly different between each group (*P* < 0.001). **b** Hepatic fECV was significantly greater in patients with DM than in nondiabetes (*P* < 0.05). No significant difference was found between nondiabetes and pDM patients or between pDM and DM patients (*P* = 0.31 and 0.09, respectively). **c** Pancreatic fECV is significantly different between each group without cirrhosis and moderate-to-severe anemia (*p* < 0.001). **d** Hepatic fECV was significantly greater in patients with DM than in nondiabetes without cirrhosis and moderate-to-severe anemia (*P* < 0.05). No significant differences were observed between nondiabetes and pDM patients or between pDM and DM patients (*P* = 0.25 and 0.19, respectively). **e** Pancreatic fECV is significantly greater in patients with DM than in nondiabetes with cirrhosis or moderate-to-severe anemia (*P* < 0.05). No significant differences were observed between nondiabetes and pDM patients or between pDM and DM patients (*P* = 0.20 and 0.44, respectively). **f** No significant differences were found in hepatic fECV among the three groups with cirrhosis or moderate-to-severe anemia (nondiabetes vs. pDM patients, *P* = 0.90; nondiabetes vs. DM patients, *P* = 0.93; pDM vs. DM patients, *P* = 0.88)

**Fig. 4:**
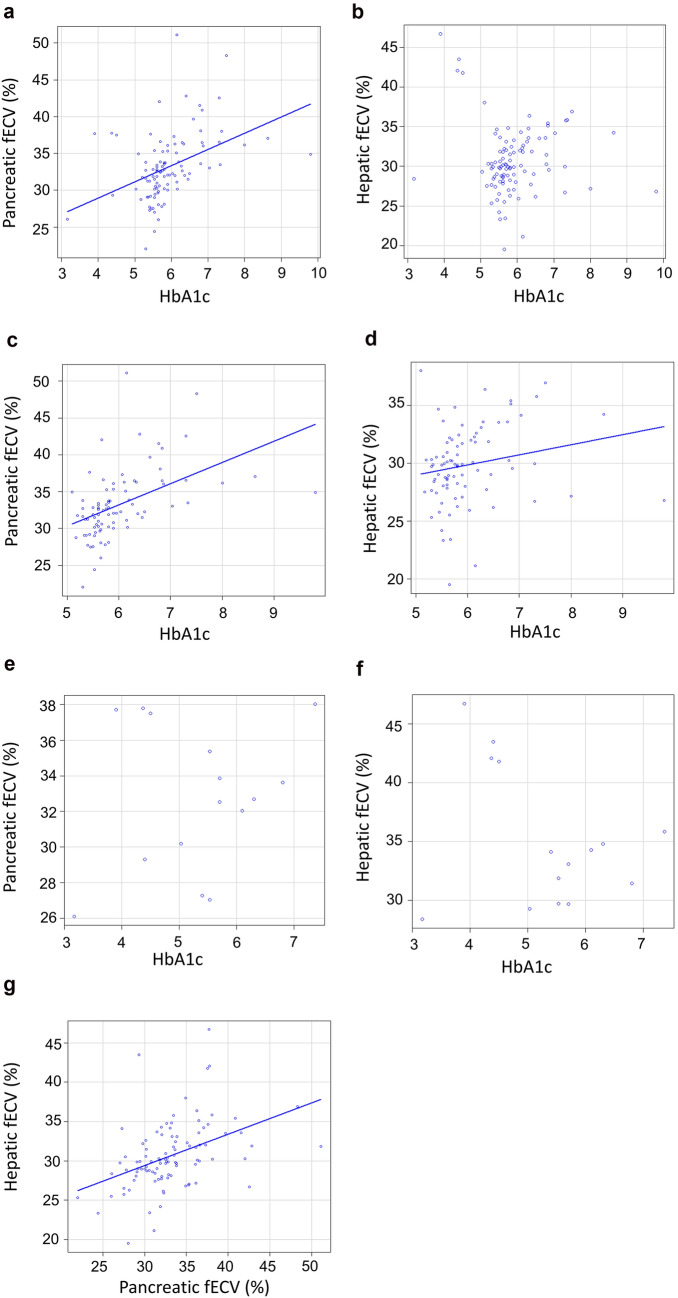
Correlations among HbA1c, pancreatic fECV, and hepatic fECV. **a** The pancreatic fECV was moderately correlated with HbA1c in all patients (r = 0.51, *P* < 0.001). **b** No correlation was found between haptic fECV and HbA1c in all patients (*P* = 0.15). **c**, **d** The pancreatic and hepatic fECV were correlated with HbA1c in patients without cirrhosis and moderate-to-severe anemia (r = 0.60, *P* < 0.001; r = 0.27, *P* < 0.05, respectively). **e**, **f** No correlation was found in patients with cirrhosis or moderate-to-severe anemia between hepatic fECV and HbA1c and between pancreatic fECV and HbA1c (*P* = 0.58 and *P* = 0.51, respectively). **g** The pancreatic fECV was moderately correlated with the hepatic fECV (r = 0.51, *P* < 0.001)

Pancreatic fECV was significantly greater among patients with DM compared with pDM or nondiabetes and in pDM compared with nondiabetes (*P* < 0.001, respectively) (Fig. 3a). Hepatic fECV was significantly greater among patients in the DM group than among those in the nondiabetes group (*P* < 0.05) (Fig. 3b). The pancreatic fECV was moderately correlated with the hepatic fECV (r = 0.51, *P* < 0.001) (Fig. 4g). The pancreatic fECV was moderately correlated with HbA1c levels (r = 0.51, *P* < 0.001) (Fig. 4a). No significant correlation was found between hepatic fECV and HbA1c (*P* = 0.15) (Fig. 4b).

**Fig. 5:**
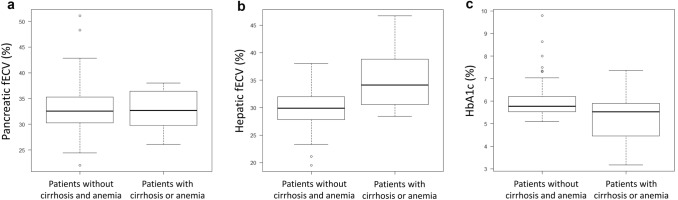
Box plot showing the comparisons of hepatic fECV, pancreatic fECV, and HbA1c levels in patients with/without cirrhosis or moderate-to-severe anemia. **a** Box plot showing the comparison of pancreatic fECV across both groups. No significant difference (*P* = 0.93) was observed, indicating similar pancreatic fECV in patients with/without cirrhosis or moderate-to-severe anemia. **b** Box plot showing a significant increase (*P* < 0.001) in hepatic fractional extracellular volume (fECV) in patients with cirrhosis or moderate-to-severe anemia as compared with those without these conditions. **c** A representation of HbA1c levels depicted through a box plot, showing significantly lower HbA1c (*P* < 0.05) in patients with cirrhosis or moderate-to-severe anemia than those without these conditions

The results of subgroup analyses for patients with and without cirrhosis and/or moderate-to-severe anemia were as follows: pancreatic fECV differed significantly in each group without cirrhosis and moderate-to-severe anemia (*p* < 0.001) (Fig. 3c). Hepatic fECV was significantly greater among patients with DM than among nondiabetes patients without cirrhosis and moderate-to-severe anemia (*P* < 0.05) (Fig. 3d). Pancreatic fECVs were significantly greater among patients with DM than among nondiabetes patients with cirrhosis or moderate-to-severe anemia (*P* < 0.05) (Fig. 3e). No significant differences were found in hepatic fECV among the three groups with cirrhosis or moderate-to-severe anemia (nondiabetes patients vs. pDM patients, *P* = 0.90; nondiabetes patients vs. DM patients, *P* = 0.93; pDM patients vs. DM patients, *P* = 0.88) (Fig. 3f). Hepatic fECV was significantly higher (*P* < 0.001) and HbA1c was significantly lower (*P* < 0.05) in patients with cirrhosis or moderate-to-severe anemia than in those without, but no significant difference was observed in pancreatic fECV (*P* = 0.93) (Fig. 5a–c).

The pancreatic and hepatic fECVs were correlated with HbA1c among patients without cirrhosis and moderate-to-severe anemia (r = 0.60, *P* < 0.001; r = 0.27, *P* < 0.05, respectively) (Fig. 4c and d). No significant correlation was found among patients with cirrhosis or moderate-to-severe anemia between hepatic fECV and HbA1c and between pancreatic fECV and HbA1c (*P* = 0.58 and 0.51, respectively) (Fig. 4e, f). No significant difference was observed in the correlation coefficients between pancreatic fECV and HbA1c in all patients vs. those among patients without cirrhosis and moderate-to-severe anemia (*P* = 0.40).

**Fig. 6:**
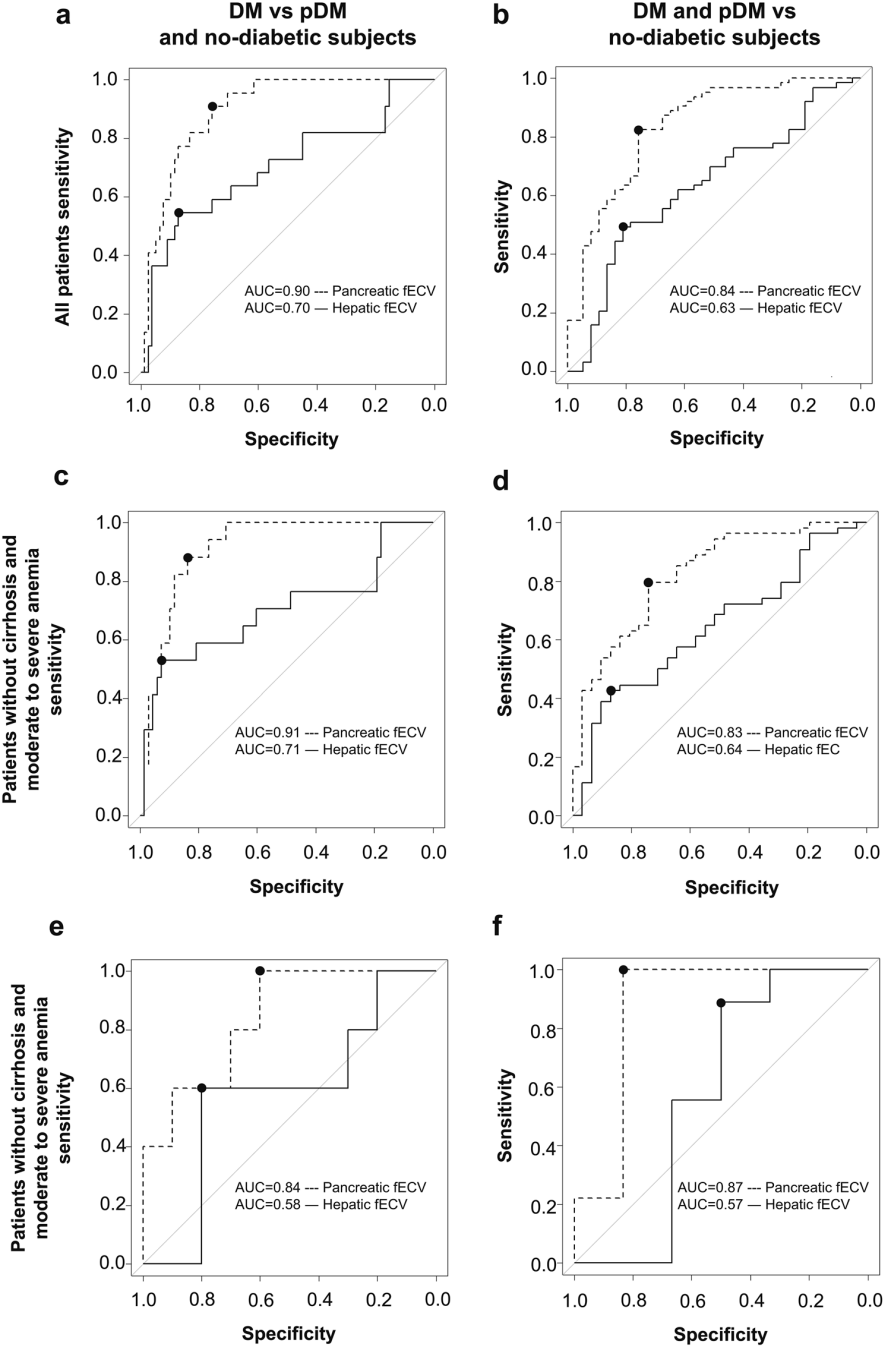
ROC curves for determining the efficacy of pancreatic and hepatic fECV in estimating patients with DM vs. pDM and nondiabetes and in DM and pDM vs. nondiabetes. Comparing the AUCs of pancreatic and hepatic fECVs in all patients **a**, **b**, in patients without cirrhosis and moderate-to-severe anemia **c**, **d**, and in patients with cirrhosis or moderate-to-severe anemia **e**, **f**

ROC curves for determining the efficacy of pancreatic and hepatic fECV in estimating patients in the DM group vs. those in the pDM and nondiabetes groups and in estimating patients in the DM and pDM groups vs. those in the nondiabetes group in all patients, patients without cirrhosis and moderate-to-severe anemia, and patients with cirrhosis or moderate-to-severe anemia are shown in Fig. 6. Cutoff values, AUC values, sensitivity, and specificity are shown in Table 3. AUC values for pancreatic fECV in estimating patients with DM vs. pDM and nondiabetes were significantly greater than hepatic fECVs in all patients, patients without cirrhosis and moderate-to-severe anemia, and patients with cirrhosis or moderate-to-severe anemia (*P* < 0.01, *P* < 0.05, and *P* < 0.05, respectively) (Fig. 6a, c, and e). The AUC value for pancreatic fECV in estimating patients with DM and pDM vs. no-diabetes was significantly greater than hepatic fECV in all patients, patients without cirrhosis and moderate-to-severe anemia, and patients with cirrhosis or moderate-to-severe anemia (*P* < 0.01, *P* < 0.01, and *P* < 0.05, respectively) (Fig. 6b, d, and f). The sensitivity for pancreatic fECV in estimating patients with DM vs. pDM and nondiabetes was significantly greater than hepatic fECV in patients without cirrhosis and moderate-to-severe anemia (*P* < 0.05). No significant differences were observed in all patients and patients with cirrhosis or moderate-to-severe anemia (*P* = 0.07 and 0.44, respectively). The sensitivity of pancreatic fECV in differentiating patients in the DM and pDM groups from those in the no-diabetes group was significantly greater than that of hepatic fECV in all patients and patients without cirrhosis and moderate-to-severe anemia (*P* < 0.01 and *P* < 0.01, respectively). No significant differences were observed in patients with cirrhosis or moderate-to-severe anemia (*P* = 0.73).

**Table 3:** ROC analysis for differentiating between patients with nondiabetes versus pDM and DM and between patients with nondiabetes and pDM versus DM

	Hepatic fECV	Pancreatic fECV	*P*-value
Patients with DM vs. pDM and nondiabetes in all patients			
Cutoff value (%)	33.5	33.5	N.A
Sensitivity (%)	54.5 (34.7–73.1)	90.9 (72.2–97.5)	0.07
Specificity (%)	87.2 (78.0–92.9)	75.6 (65.1–83.8)	0.08
AUC (95% CI)	0.70 (0.56–0.84)	0.90 (0.84–0.96)	< 0.01
Patients with DM vs. pDM and nondiabetes in patients without cirrhosis and moderate-to-severe anemia			
Cutoff value (%)	33.5	34.9	N.A
Sensitivity (%)	52.9 (31.0–73.8)	83.8 (59.0–93.8)	< 0.05
Specificity (%)	92.6 (83.9–96.8)	88.2 (78.5–93.9)	0.11
AUC (95% CI)	0.71 (0.54–0.87)	0.91 (0.85–0.97)	< 0.05
Patients with DM vs. pDM and nondiabetes in patients with cirrhosis or moderate-to-severe anemia			
Cutoff value (%)	35.9	32.5	N.A
Sensitivity (%)	60.0 (23.1–88.2)	100 (56.6–100)	0.44
Specificity (%)	80.0 (49.0–94.3)	60.0 (31.3–83.2)	0.62
AUC (95% CI)	0.58 (0.24–0.92)	0.84 (0.63–1.00)	< 0.05
Patients with DM and pDM vs. nondiabetes in all patients			
Cutoff value (%)	31.4	31.9	N.A
Sensitivity (%)	49.2 (37.3–61.2)	82.5 (71.3–90.0)	< 0.01
Specificity (%)	81.1 (65.8–90.5)	75.7 (59.9–86.7)	0.72
AUC (95% CI)	0.63 (0.52–0.75)	0.84 (0.75–0.92)	< 0.01
Patients with DM and pDM vs. nondiabetes in patients without cirrhosis and moderate-to-severe anemia			
Cutoff value (%)	31.5	31.9	N.A
Sensitivity (%)	42.6 (30.3–55.8)	79.6 (67.1–88.2)	< 0.01
Specificity (%)	87.1 (71.1–94.9)	74.2 (56.8–86.3)	0.22
AUC (95% CI)	0.64 (0.52–0.76)	0.83 (0.73–0.92)	< 0.01
Patients with DM and pDM vs. nondiabetes in patients with cirrhosis or moderate-to-severe anemia			
Cutoff value (%)	31.4	32.0	N.A
Sensitivity (%)	88.9 (56.5–98.0)	100 (67.6–100)	0.73
Specificity (%)	50.0 (18.8–81.2)	83.3 (43.7–97.0)	0.48
AUC (95% CI)	0.57 (0.19–0.96)	0.87 (0.61–1.00)	< 0.05

*fECV* extracellular volume fraction, *AUC* area under the receiver operating characteristic curve, *CI* confidence interval, *pDM* prediabetes, *DM* diabetes mellitus, *N.A* not applicable

## Discussion

This study revealed that pancreatic fECV was moderately correlated with hepatic fECV (r = 0.51, *P* < 0.001). Pancreatic fECV and hepatic fECV reflect the degree of pancreatic fibrosis and liver fibrosis, respectively [11–15], suggesting the correlation between progression of fibrosis in these organs. Pancreatic stellate cells play a central role in pancreatic fibrosis. Watanabe et al. reported that pancreatic stellate cells are activated by increased pancreatic tissue pressure [28]. Increased pancreatic tissue pressure due to portal hypertension in chronic liver disease was speculated to activate pancreatic stellate cells and promote pancreatic fibrosis [29].

The most reliable assessment method of organ fibrosis is a pathological evaluation using techniques such as biopsies with histopathology. However, biopsies are not easily performed, especially in the pancreas, which exists deep in the body, because they are invasive. Blood markers, US elastography, and MR elastography have been established as noninvasive methods for evaluating fibrosis in the liver [9, 10]. Conversely, no noninvasive evaluation method for fibrosis has been established in the pancreas. Recently, the evaluation of fibrosis by fECV using contrast-enhanced CT has been reported, and this analytical method can be used to evaluate fibrosis in the liver and pancreas [11–15]. This method is advantageous because the fECV of the liver and pancreas can be calculated from the image data of upper abdominal CT imaging. Comprehensive management of fibrosis in the liver and pancreas may be required because the progression of fibrosis in these organs is correlated. Therefore, fECV analyses that allow the simultaneous assessment of both pancreatic and hepatic fibrosis may be clinically useful.

The present study revealed a correlation between the pancreatic and hepatic fECVs and the severity of DM. Per previous reports, pancreatic or hepatic fibrosis is correlated with the severity of DM [6–8, 30–33], in line with the present study. Regarding the correlation between pancreatic fECV and diabetes severity, pancreatic ECV has been reported to serve as a potential imaging biomarker for the evaluation of pancreatic fibrosis, which leads to impaired glucose tolerance, per fECV analyses using contrast-enhanced MRI [34]. A previous study investigated the association between fECV, as gauged by dual-energy CT, and DM [35]. Yet, contrary to our research and the outcomes reported by Noda Y et al., a correlation between pancreatic fECV and HbA1c was not observed in this study [34, 35]. Such a discrepancy could be attributed to the fact that two-thirds of the patients were diagnosed with cirrhosis, which may have led to the lack of correlation. Compared to MRI, contrast-enhanced CT has several advantages such as its ease of examination and paucity of image artifacts. No study has reported the correlation between hepatic fECV and diabetes regarding the liver. Our results suggest that the fECV analysis of contrast-enhanced CT is an imaging biomarker of pancreatic and hepatic fibrosis associated with DM severity.

HbA1c is the gold standard for measuring long-range glycemic control in patients with DM. However, cirrhosis and moderate-to-severe anemia have been reported to have lower HbA1c levels in patients with DM [20, 21], which makes HbA1c a poor indicator of DM in these patients. Hepatic fECV was significantly larger in patients with cirrhosis or moderate-to-severe anemia than in those without, which might be attributable to liver fibrosis caused by liver cirrhosis. Simultaneously, the significant decrease in HbA1c can be associated with liver cirrhosis. As for pancreatic fECV, no substantial differences were detected, which aligns with the results of the study by Kameda et al. [35]. This may imply that the influence of cirrhosis and anemia on pancreatic fECV is less when indicating DM severity. The absence of a significant correlation between hepatic fECV and HbA1c may be due to the cohort’s inclusion of patients with cirrhosis and moderate-to-severe anemia. The correlation between hepatic fECV and HbA1c appeared in patients without cirrhosis and moderate-to-severe anemia. Moreover, the correlation coefficient between pancreatic fECV and HbA1c increased more in patients without cirrhosis and moderate-to-severe anemia than in all patients. Additionally, ROC analyses were also performed to determine the feasibility of using pancreatic and hepatic fECV to stratify DM severity. Both pancreatic and hepatic fECVs yielded satisfactory AUCs, which may be useful for stratifying DM severity. However, FPG is useful for assessing DM severity, and the clinical use of fECV may be controversial.

This study has several limitations. First, blood was not drawn on the day of the CT scan in some cases; however, recent studies revealed no significant difference between hematocrit values obtained on the day of the imaging study and fECV calculated from hematocrit values obtained on another day [36]. This indicates that the day on which the hematocrit value was obtained does not significantly affect the measurement of fECV. Second, concerning imaging techniques, the 5-mm slice thickness for fECV measurements may not be optimal for measuring pancreatic fibrosis. Third, DM stratification may be insufficient because a 75-g 2-h oral glucose tolerance test was not performed. Fourth, a correlation between fECV and fibrosis has been reported in the liver and pancreas, although there is no histopathological evidence. Fifth, our research approach did not account for confounding factors, such as venous congestion, liver fat, and intralobular pancreatic fat. Sixth, our timing of the equilibrium phase may not allow sufficient time for the contrast medium to equilibrate. Yoon et al. reported that an equilibrium phase of 180 s after contrast administration, used for estimating ECV, represents a good compromise between clinical workflow and technical success [13]. In our study, the equilibrium phase occurred approximately 204 s after contrast administration, and no patients exhibited a portal vein CT value > 10 Hounsfield units higher than the aortic CT value. Seventh, two CT scanner vendors were used in the current study. The performance of ECV measured by CT scanners from other vendors has not been assessed. Finally, this was a single-center retrospective observational study. Multicenter prospective studies are needed to determine the usefulness of fECV in stratifying DM.

In conclusion, the findings of this study highlighted the close association between hepatic fECV, pancreatic fECV, and pDM/DM. We found that fECV, in addition to HbA1c, is a valuable proven clinical tool for assessing the status of diabetes. For patients with unreliable HbA1c measurements due to various conditions, such as cirrhosis or anemia, pancreatic fECV should be considered.

## Abbreviations

fECV: Extracellular volume fraction
SSIM: Sure subtraction iodine mapping
ROC: Receiver operating characteristic
ROI: Region of interest
AUC: Area under the curve
DM: Diabetes mellitus
pDM: Prediabetes mellitus
FPG: Fasting blood glucose
HbA1c: Hemoglobin A1C
